# Does Size Matter? Mate Choice in Two Lekking Flies

**DOI:** 10.1093/jisesa/ieaa019

**Published:** 2020-04-11

**Authors:** Marco Tulio Tejeda, José Arredondo, Francisco Díaz-Fleischer, Diana Pérez-Staples

**Affiliations:** 1 Subdirección de Filtrado Genético, Programa Operativo Moscamed Acuerdo Sader-IICA. Planta Moscamed, Chiapas, México; 2 Departamento de Biología, Ecología y Comportamiento, Desarrollo de métodos, Programa Moscafrut (SADER-SENASICA), Camino a los Cacaotales S/N, Chiapas, México; 3 INBIOTECA, Universidad Veracruzana. Veracruz, México

**Keywords:** female choice, sterile insect technique, lek, Tephritidae

## Abstract

For insect pests controlled through the Sterile Insect Technique (SIT), which consists in the mass production, sterilization, and release of target insects into affected areas, sterile male mating success with wild females is the key that will reduce population levels in the next generation. Male size is assumed to be important for mating success, but often without any concrete evidence or confounded by other parameters. Here, we evaluated male size and its interaction with male origin (laboratory or wild) on female choice for two lekking species controlled through SIT, *Anastrepha obliqua* (Macquart) and *Anastrepha ludens* (Loew) (Diptera: Tephritidae). In field cages, we tested wild females mating with: large wild males competing against small wild males; large wild males against small laboratory-sterile males; and large laboratory-sterile males against small wild males. We found evidence of large male advantage for *A. obliqua* but no effect of male size on mating competitiveness for *A. ludens*. For *A. obliqua* large wild males had a greater mating success over small laboratory males, yet large laboratory males secured a similar amount of copulations than small wild males. For *A. ludens*, there was no effect of male size on mating success. We discuss why *A. obliqua* is sensitive to size and origin while no effect was seen in *A. ludens*. SIT programs should not assume that male mating success is dependent on a large size. Alternatively, when an advantage exists for large males, mass-rearing programs should stride to produce and release large males.

## Abstract in Spanish

Para los insectos plaga controlados a través de la Técnica del Insecto Estéril (TIE), la cual consiste en la producción masiva, esterilización y liberación de insectos en las áreas afectadas, el éxito de apareamiento del macho estéril con hembras silvestres es la clave para reducir los niveles de población en la siguiente generación. El tamaño del macho puede ser importante para el éxito de apareamiento, sin embargo, para muchas especies no tenemos evidencia concreta o está confundida por otros parámetros. Aquí evaluamos el tamaño del macho y su interacción con su origen (laboratorio o silvestre) en la elección femenina de *Anastrepha obliqua* y *Anastrepha ludens*, dos especies que forman leks y que son controladas a través de la TIE. En jaulas de campo estudiamos el apareamiento de hembras silvestres con: machos silvestres grandes que compiten contra machos silvestres pequeños; machos silvestres grandes contra machos estériles pequeños y machos grandes estériles contra machos pequeños silvestres. Encontramos evidencia de una ventaja en la competitividad sexual para los machos grandes en A. obliqua, pero ningún efecto del tamaño del macho para A. ludens. Para A. obliqua, los machos silvestres grandes tuvieron un mayor éxito de apareamiento que los machos de laboratorio pequeños. Sin embargo, los machos de laboratorio grandes obtuvieron una cantidad similar de cópulas que los machos silvestres pequeños. Para A. ludens, no hubo efecto del tamaño del macho en el éxito del apareamiento. Discutimos por qué A. obliqua es sensible al tamaño y al origen, mientras que no se observó ningún efecto en A. ludens. Los programas que implementan la TIE no deben asumir que el éxito del apareamiento depende de un tamaño grande del macho. Alternativamente, cuando existe una ventaja para los machos grandes, los programas de cría en masa deben producir y liberar machos grandes.

Male mating success in lekking species depends on male–male competition for calling sites and on female choice based on complex precopulatory courtship (Höglund and Alatalo 1995, Shelly 2018). For tephritid flies that form leks, males compete for calling sites on the underside of host and nonhost trees and then emit pheromones and court females that arrive at the lek sites (Aluja et al. 2000). Females are thought to compare males, and be highly selective in their mating decisions, generating a large variance in male mating success (Shelly and Whittier 1997, Shelly 2018). Programs that use the sterile insect technique (SIT) to control pest tephritids must account for female choice for sterile males at these lek sites. The SIT consists in the mass production, sterilization usually by irradiation of a cobalt source, and release of sterile insects into affected areas, where sterile males are expected to mate with wild females and transfer dominant lethal mutations that render the female sterile (Knipling 1955). Thus, mating preferences of wild females play a critical role in programs where the SIT is applied, yet despite many years of research, there is still no consensus on what phenotypes females prefer.

Female tephritids controlled through the SIT must choose to mate between wild or sterile males, and male size is often thought to be a major factor affecting female choice. Yet, in tephritids of economic importance, there is conflicting evidence for size effects in mating preferences, and the evidence for positive size assortment is not clear cut (Shelly 2018). For example, for wild Mexican fruit fly, *Anastrepha ludens* (Loew) tested in field cages, larger males were more successful in mating but only when males were calling in pairs, perhaps allowing for female comparison. For the guava fruit fly *Anastrepha striata* Schiner, larger males were more likely to mate repeatedly than medium or small males. However, for both species, there was no effect of male size on participation in leks or mating success (Aluja et al. 2008). Also, in a laboratory study for wild *A. ludens* and the West Indies fruit fly, *Anastrepha obliqua* (Macquart), there was no effect of male size on mating success (Aluja et al. 2009). Thus, for these three *Anastrepha* species, there is no strong evidence of a competitive advantage for bigger males. In contrast, for other *Anastrepha* species such as the Caribbean fruit fly *Anastrepha suspensa* (Loew), females prefer larger males and in the South American fruit fly *Anastrepha fraterculus* (Wiedemann), males with longer wing lengths and thorax size were more successful at mating (Burk and Webb 1983, Sciurano et al. 2007). Furthermore, in a field cage study for *A. fraterculus*, a positive correlation between eye length and copulatory success was found (Segura et al. 2007).

There is also conflicting evidence for the Mediterranean fruit fly *Ceratitis capitata* (Wiedemann). For example, both wild and laboratory females have been found to prefer larger males (Churchill Stanland et al. 1986, Orozco and López 1993, Blay and Yuval 1997, Rodriguero et al. 2002a). In addition, males of this species were more successful in obtaining copulations if they had a larger thorax and narrower face (Rodriguero et al. 2002b). In contrast, other studies have found no relationship between size and mating success (Whittier et al. 1994, Whittier and Kaneshiro 1995, Norry et al. 1999, Shelly 2000). The relative effects of strain and size are also variable. In some studies with *C. capitata* both laboratory and wild females have preferred large males, while in others wild females prefer wild males over laboratory males independent of size, and laboratory females prefer to mate with larger males (Câmara de Aquino and Joachim-Bravo 2014).

Evidence of size effects in tephritids is also confounded by the fact that results from laboratory studies may not match more natural conditions in field cages. For example, in the Queensland fruit fly *Bactrocera tryoni* (Froggat), no size effects were observed in mating studies under laboratory conditions (Pérez-Staples et al. 2007, 2008, Prabhu et al. 2008). However, a recent field cage study revealed that larger males were more successful at mating, and within the large male group, successful males had larger wings compared to males that did not mate (Ekanayake et al. 2017). Thus, field cage studies, where males compete under a more natural setting could be more informative than laboratory results. Clearly, female choice for male size of either wild or laboratory males still needs further study. If there are size preferences of wild females for sterile males, this has important implications for mass-rearing production and release decisions.

In the present study, we evaluated the effect of male size on wild female mating preferences for two lekking species of tephritids that are currently controlled through SIT, *A. ludens* and *A. obliqua*. Using a sexual competition test with wild females in field cages, we evaluated the hypothesis that sterile laboratory-reared males of different sizes are equally competitive. In addition, and because of their importance in pest management programs, we also assessed the interactions between wild and laboratory-sterile males in order to establish if male origin is a factor that affects size preferences in mate choice.

## Materials and Methods

### Insects

Wild and laboratory insects were used for both *A. ludens* and *A. obliqua*. Wild insects were collected from infested fruits in the field. For *A. ludens,* fruits of *Citrus aurantium* (L.) (Sapindales: Rutaceae) Macfady (bitter orange) were collected in the surroundings of Metapa de Domínguez, Chiapas, Mexico. For *A. obliqua,* infested fruits of tropical plum, *Spondias purpurea* (L.) (Sapindales: Anacardiaceae), were collected in the surroundings of Comalapa, Chiapas. In both cases, infested fruits were transported to the laboratory in trays with a layer of vermiculite as pupation substrate. Mature larvae were separated from the fruit and placed in moist vermiculite to stimulate pupation. Laboratory insects were obtained from the mass-rearing colonies of the Moscafrut biofactory at Metapa de Domínguez, Chiapas (Domínguez et al. 2010). The standard bisexual strain was used for laboratory flies of both species.

Two days before eclosion and under hypoxic conditions, laboratory insects were subjected to a standard sterilizing dose of 80 Gy with a ^60^Co irradiator (model GB-127, Nordion International Inc., Ottawa, Ontario, Canada).

### Insect Size

Pupal diameter was taken as the measure of insect size. A pupae-sorting machine was used to standardize insect size throughout the experiments (FAO/IAEA/USDA 2014). The machine relies on two distance-adjustable cylinders to create an increasing gap canal that separates pupae by their transverse plane diameter. The sorting machine isolates ten discrete diameter classes. A batch of >4,000 wild individuals was used to standardize the separating canal of the sorting machine (Table 1). The standardization followed two criteria: 1) the 6th diameter class should have the most pupae, and 2) each of the 2nd and 10th diameter classes should contain less than 5% of total pupae. A normal distribution was established where: the 2nd class had the smallest individuals, the 6th had the average individuals, and the 10th class had the largest individuals. All pupae for each fly species, wild or laboratory, were subjected to the standardization of the first wild batch.

**Table 1.** Size diameter intervals of wild and laboratory-reared pupae used to evaluate the effect of male size on female mate choice of two lek-forming fly species

	Pupae size (mm)					
Species	Small males		Large males		Females	
	min	max	min	max	min	max
*Anastrepha obliqua*	1.70	1.87	2.26	2.49	2.04	2.15
*Anastrepha ludens*	2.19	2.40	2.72	2.92	2.51	2.61

The values were based on the size of *A. obliqua* reared on *Spondias purpurea* and *A. ludens* reared on *Citrus aurantium.*

Three size categories were then established: small, average, and large flies. Small flies were those emerged from pupae of the third and fourth diameter class. Average size flies were those emerged from pupae of the sixth diameter class. Large flies were those emerged from pupae of the eighth and ninth diameter class. For both species, the corresponding pupal diameter range of these categories is presented in Table 1.

Extremely small and extremely large pupae (2nd and 10th classes) were discarded to avoid bias (poor nutrition, development malfunction). Also, in order to be confident of size differences between large and small males, diameter classes adjacent to the average size class (fifth and seventh classes) were also discarded. All females used for the experiments were taken only from the average size category (sixth diameter class), while males were only taken from the small or large size category (third to fourth or eighth to ninth diameter classes, respectively).

The gap clearances within the two cylinders were measured at several points along the separating path with the help of a 26-blade master feeler gauge (Powerbuilt model 648717, Briggs & Stranton Corporation, Wisconsin). A linear regression was then fitted for each species. For *A. obliqua*, pupal diameter (mm) (Y) corresponded with sorting class (X) following the equation *Y= 0.1122X+1.4228*; while for *A. ludens*, pupal diameter (mm) corresponded with sorting class following *Y= 0.1057X + 1.8738*.

### Insect Handling and Enclosure Conditions

Upon emergence, insects were sorted by sex and kept in wooden-framed mesh cages (30 × 30 × 30 cm), provided ad libitum with water and a standard diet (sugar and hydrolyzed yeast (MP Biomedicals, LLC, California) at a 3:1 ratio). Wooden cages with flies were maintained in a L12:D12 photoperiod, 60–80% relative humidity, and at 25 ± 2°C room temperature. To avoid possible stress due to overcrowding, wild flies were maintained at approximately 100 adults per cage. For laboratory flies, 150–200 individuals were held per cage.

Two days before observations in the field cages, males were marked using nontoxic food colorants (McCormick Assorted Food Colors, Maryland) dissolved in diet. Approximately 0.12 ml (two drops) of red or blue colorant were added to 150 g of adult diet (as above). To avoid any behavioral bias caused by marking, colors were switched between replicates and the effect of alternate marking was analyzed.

On the day of the field cage test, insects were placed in smaller cages (15 × 15 × 15 cm) to facilitate transportation and release. Adults were sexually mature when released, wild insects were 14–16 d old, while laboratory insects were 9–11 d old (FAO/IAEA/USDA 2014).

### Field Cages

Observations were carried out in field cages of 3 m in diameter by 2 m high with host trees placed inside (Calkins and Webb 1983). Orange and mango trees in pots were placed along the inner perimeter and in the center of the cage. On the day of the test, insects were released into the cages according to the sexual calling period of each species: *A. obliqua* was released at 06:00, while *A. ludens* was released at 16:00 h (Aluja et al. 2000). In both cases, males were released 1 h before females to allow the establishment of territories and lek formation. Observations in field cages ended at 11:00 a.m. for *A. obliqua* and at sunset for *A. ludens*. Each field cage was constantly monitored, mating pairs were gently collected, and male type (small or large) was registered.

### Male Mating Success According to Size

To evaluate the effect of male size on mate choice, we released large and small males together with wild virgin females in a 2:1 male–female ratio. For both species, a ratio of 1:1:1 (1 wild female: 1 small male:1 large male) was used in all field cages. For *A. ludens*, a density of 75 individuals per field cage (25 wild females, 25 small males, and 25 large males) was used in all field cages. For *A. obliqua*, density varied between 75 and 54 individuals per field cage (1:1:1 ratio was maintained) according to available pupae.

Size according to male origin was evaluated in three experiments. The first experiment consisted of large wild males (Lw) competing with small wild males (Sw). For the second experiment, large wild males (Lw) were released together with small laboratory (Sl) males. Finally, the third experiment consisted in large laboratory males (Ll) competing with small wild (Sw) males. Each experiment was repeated 7–11 times with 6 different cohorts of flies.

### Statistical Analysis

To determine if there was an overall effect of male size on mate choice, total mating frequency of each male size (copulations with small or large males) was obtained for each treatment and was tested against the proportion expected for equal sexual competitiveness (1:1) with a chi-squared 3 × 2 contingency table (3 treatments or experiments × 2 male sizes, i.e., total effect of male size) (α = 0.05).

To examine if male origin (laboratory or wild) is a source of bias for size preferences in mate choice, the proportion of copulations between large wild and small laboratory males (Lw-Sl) was contrasted against the proportion of copulations between large laboratory versus small wild males (Ll-Sw) using a chi-squared 2 × 2 contingency table (2 treatments × 2 male size categories).

To evaluate size mating preferences within treatment, copulation frequency of small and large males was contrasted against the 1:1 theoretical proportion expected for equal sexual competitiveness between males using a chi-squared test, per treatment (Lw-Sw, Lw-Sl, Ll-Sw).

To determine if color marking affected the mating pattern, the proportions of copulas secured by male size categories was contrasted between alternate marking combinations (i.e., large&red-small&blue vs large&blue-small&red) was analyzed by a 2 × 2 contingency chi-squared table (2 male size categories × 2 marking combinations), per treatment and species.

## Results

### Anastrepha obliqua

**Fig. 1.**
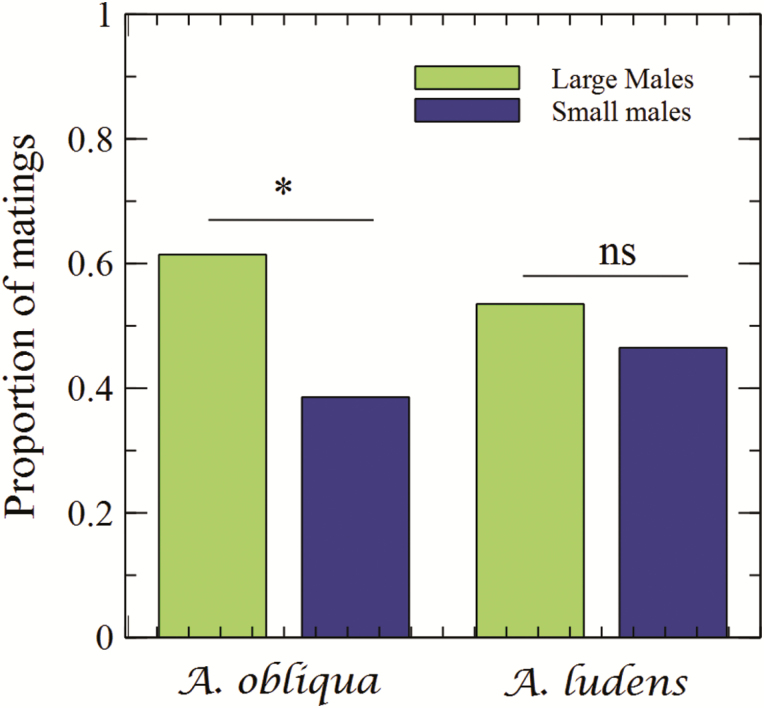
Proportion of matings obtained by *Anastrepha obliqua* and *Anastrepha ludens* males of different sizes competing for wild females in field cages. Asterisk above column denotes a significant difference (α = 0.05).

Three hundred and six copulations were obtained out of 600 possible pairs in 27 field cages. In total, large males secured more copulations than small males (Fig. 1). Copulation frequency of large males was significantly higher than the expected under the assumption of equal competitiveness between large and small males (χ ^2^ = 20, df = 2, *P* < 0.0001).

**Fig. 2.**
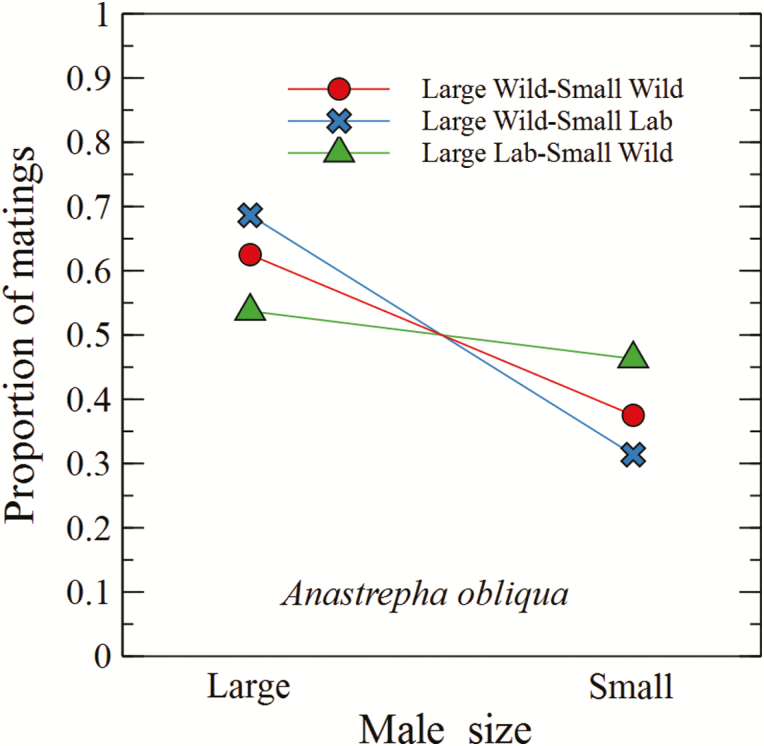
Proportion of matings obtained by sterile or wild *Anastrepha obliqua* males of different sizes competing for wild females in field cages. Significant interactions between male origin and size were found when large wild males competed against small laboratory males and when large wild males competed against small wild males.

There was an effect of male origin on the proportion of copulations with wild females (Lw-Sl vs Ll-Sw; χ ^2^ = 4.9, df = 1, *P* = 0.02). Large wild males had more matings when competing against small laboratory males (Lw-Sl) (χ ^2^ = 14.16, df = 1, *P* < 0.0001). However, there was no significant effect of size when small wild males competed with large laboratory males (Ll-Sw) (χ ^2^ = 0.59, df = 1, *P* = 0.44; Fig. 2). When only wild males, large or small, competed for matings (Lw-Sw treatment), the proportion of copulations did not fit the expected theoretical proportion (1:1) for the assumption of equal competitiveness between males (χ ^2^ = 6.0, df = 1, *P* = 0.01), with an advantage for large males (Table 2).

**Table 2.** Copulas obtained by males of contrasting sizes (large vs small) and from different origin (wild or laboratory-sterile) when competing for matings with wild females

	Size		Field cages evaluated	Total copulations		χ ^2^	*P*-value
	Large males	Small males		Large males	Small males		
*A obliqua*							
	Wild	Wild	7	60	36	6.00	**0.01**
	Wild	Laboratory	10	70	32	14.16	**<0.001**
	Laboratory	Wild	10	58	50	0.59	0.44
			27	188	118	20.75	**<0.0001**
*A. ludens*							
	Wild	Wild	10	60	49	1.11	0.29
	Wild	Laboratory	11	52	46	0.37	0.54
	Laboratory	Wild	11	71	64	0.36	0.55
			32	183	159	1.84	0.40

Values presented for two lek-forming fly species, *Anastrepha obliqua* and *Anastrepha ludens*.

For each row, the observed frequency was contrasted against the 1:1 proportion expected for equal sexual competitiveness between large and small males by a chi-squared test (α = 0.05). Values in bold are statistically significant.

### Anastrepha ludens

Three hundred and forty-two copulations were obtained out of 800 possible pairs in 32 field cages. In total, large males secured 183 copulations, while small males secured 159. Even though large males obtained numerically more copulas than small males, there was no significant difference in the proportion of matings obtained between large and small males (χ ^2^ = 1.8, df = 2, *P* = 0.39) (Fig. 1). Thus, an advantage for large males was not supported statistically. We did not find any significant difference between the proportion of copulations between large and small males for any size treatment (Lw-Sw, Lw-Sl, Ll-Sw) (Table 2).

Male origin (laboratory-wild) did not affect the proportion of copulations. There was no significant difference in the proportion of copulations secured by males between the Lw-Sl and Ll-Sw treatments (χ ^2^ = 0.005, df = 1, *P* = 0.94), indicating that mating success was not affected by male origin.

### Food Colorant Marking

Food color marking had no effect on copula frequency for *A. obliqua*: Lw-Sw (*n* = 96, χ ^2^ = 0.5, df = 1, *P* = 0.46); Lw-Sl (*n* = 102, χ ^2^ = 2.98, df = 1, *P* = 0.1); Ll-Sw (*n* = 108, χ ^2^ = 0.46, df = 1, *P* = 0.49). Likewise, for *Anastrepha ludens*, there was no significant effect of color marking on copula frequency: Lw-Sw (*n* = 109, χ ^2^ = 1.6, df = 1, *P* = 0.2); Lw-Sl (*n* = 98, χ ^2^ = 0.23, df = 1, *P* = 0.63); Ll-Sw (*n* = 135, χ ^2^ = 1.02, df = 1, *P* = 0.31).

## Discussion

A successful SIT program will depend ultimately on sterile males being successful at intersexual competition. In particular, for species that form leks, such as *A. ludens* and *A. obliqua,* selective pressures and variance in mating success is high due both to male–male competition for adequate calling sites as well as female choosiness (Shelly 2018). Here, we show, in seminatural field cage conditions, that male size and origin (laboratory reared or wild) affected *A. obliqua* but not *A. ludens* mating success. Our results for *A. ludens* coincide with previous studies on wild flies in field cages, where no effect of male size (thorax length) was found in mating success (Robacker et al. 1991), while previous laboratory studies on wild males of both of these species did not find an effect of pupal size on mating success (Aluja et al. 2008, 2009).

A large male mating advantage was seen for *A. obliqua* when large wild males competed against small laboratory-sterile males, and when large wild males competed against small wild males in field cages. Likewise, for the olive fruit fly *Bactrocera oleae* (Rossi), albeit from laboratory studies, large wild males were more successful at mating than smaller males (Benelli et al. 2016). Similarly, large wild males outcompete small males for matings in *C. capitata* (Kaspi et al. 2000), and specifically a preference for large male thorax size but narrow faces has been found for both laboratory and wild females (Rodriguero et al. 2002b). For the genetic sexing strain of *A. ludens* (Tapachula-7), a continuous selection for males that were successful at mating resulted in an increase in larval and pupal weight after four generations (Quintero-Fong et al. 2016). This result suggests that selection for mating success results in bigger males, which could be attributable to intra or intersexual competition, genetic drift, or other environmental factors (Gómez Cendra et al. 2014). While none of these studies have compared wild to sterile males, they do imply that male size may be important in certain contexts.

For *A. obliqua,* when large sterile males competed against smaller wild males there was no significant effect of size. This suggests that in field conditions, sterile *A. obliqua* males will be competitive against wild males but only when wild males are smaller. This scenario would be very positive for SIT if large males are mass-produced, sterilized and released. Indeed for *A. fraterculus*, laboratory flies have been found to be bigger than wild flies (Gómez Cendra et al. 2014). Sterile males may face different sized competitors throughout the year. For example, in *C. capitata*, the smallest wild individuals were recovered from traps in the summer compared to the winter months (Navarro-Campos et al. 2011). Sexual selection pressure for sterile males will not be the same throughout the year, because wild male size will vary depending on environmental conditions, such as temperature, humidity, host availability, and developmental time. While we do not know for *A. obliqua* how the size of wild males may vary throughout the year, certainly native host availability of tropical plums *Spondias* spp. is brief and explosive (Díaz-Fleischer and Aluja 2003), and in areas where sterile *A. obliqua* are released, wild males may be smaller during the dryer winter months when their native host is not available.

The natural history of both these species may explain why a size and origin effect was found in *A. obliqua* but not *A. ludens.* Both species exhibit similar lek sizes of two to six grouped males for *A. ludens* (Mangan 1997, Robacker et al. 2003) and two to eight males for *A. obliqua* (Aluja et al. 1983). However, the overall abundance of *A. obliqua* leks is greater than for *A. ludens* (Aluja et al. 1983), while *A. obliqua* females tend to remate less frequently than *A. ludens* females (da Silva et al. 1985, Aluja et al. 2009). Thus, a single mating may be more important for *A. obliqua* females, which may be more choosy discriminating against small males. Their frequent lekking behavior would give them more opportunity for comparison between males than for *A. ludens*, where males can also call individually and not only at lek sites.

In conclusion, size was a more important parameter for *A. obliqua* sterile males in terms of securing matings with wild females than for *A. ludens,* while large wild males will outcompete small sterile males, large sterile males can compete successfully against small wild males. Thus, program managers should pay particular attention to this quality control parameter for *A. obliqua*, ensuring that only large males are produced and released. The fact that no size effects were observed for *A. ludens* suggests that it is important not to assume that male mating success is dependent on a large size for all pest tephritids. For *A. ludens*, other factors such as male–male competition, lek site selection (e.g., Robacker et al. 1991) and pheromone quality, may be more important determinants of male copulatory success.
