# Corrigendum to “Does Size Matter? Mate Choice in Two Lekking Flies”

**DOI:** 10.1093/jisesa/ieaa037

**Published:** 2020-06-10

Correction of “Tejeda M. T., J. Arredondo, F. Díaz-Fleischer, and D. Pérez-Staples. 2020. Does Size Matter? Mate Choice in Two Lekking Flies. J. Insect. Sci.”

DOI: 10.1093/jisesa/ieaa019

In the original published version of this article, the affiliation for Dr. Perez-Staples was incorrect. The authors regret this error.

